# Differences in Genetic Background Contribute to *Pseudomonas* Exotoxin A-Induced Hepatotoxicity in Rats

**DOI:** 10.3390/toxins9070224

**Published:** 2017-07-15

**Authors:** Chien-Chao Chiu, Yu-Chih Wang, Wen-Ching Huang, Yi-Hsun Chen, Shao-Wen Hung, Yen-Te Huang, Hsiao-Li Chuang, Yi-Chih Chang

**Affiliations:** 1 Animal Technology Laboratories, Agricultural Technology Research Institute, Miaoli 350, Taiwan; chiu2295@yahoo.com.tw (C.-C.C.); 1032169@mail.atri.org.tw (S.-W.H.); 2 Graduate Institute of Veterinary Pathobiology, College of Veterinary Medicine, National Chung Hsing University, Taichung 402, Taiwan; joelovefu@gmail.com (Y.-C.W.); u9423201@gmail.com (Y.-H.C.); 3 Department of Exercise and Health Science, National Taipei University of Nursing and Health Sciences, Taipei 112, Taiwan; magicpica521@gmail.com; 4 National Laboratory Animal Center, National Applied Research Laboratories, Taipei 115, Taiwan; evan@nlac.narl.org.tw (Y.-T.H.); p650214@nlac.narl.org.tw (H.-L.C.); 5 Department of Medical Laboratory Science and Biotechnology, China Medical University, Taichung 404, Taiwan

**Keywords:** *Pseudomonas* exotoxin A, genetic background, hepatotoxicity, massive necrosis

## Abstract

*Pseudomonas aeruginosa* exotoxin A (PEA) causes severe hepatotoxicity in experimental animals and is useful in investigations of immune-mediated liver injury. However, strain differences in the sensitivity to PEA-induced hepatotoxicity in rats remains be elucidated. In this study, we determined the severity of PEA-induced hepatotoxicity in six genetically different rat strains. Male LE (Long Evans), Wistar, F344, WKY, BN/SsN and LEW rats were administered a single intravenous injection of PEA (20 μg/kg). Significantly elevated serum ALT and AST levels, massive necrosis and hemorrhage, and numerous TUNEL-positive hepatocytes were observed in BN/SsN rats. In contrast, low levels of ALT and AST as well as mild changes in liver histopathology were observed in Wistar and F344 rats. Moderate levels of hepatic injuries were observed in LE, WKY, and LEW rats. Pro-inflammatory cytokines including TNF-α, IL-2 and IL-6 serum levels were markedly increased in BN/SsN rats compared to Wistar and F344 rats. However, the hepatic levels of low density lipoprotein receptor-related protein (LRP), which functions as the PEA receptor, were not significantly different in each strain. Taken together, we suggest that BN/SsN is the most sensitive rat strain, whereas Wistar and F344 were the most resistant rat strains to PEA-induced liver damage. The different genetic background of rat strains plays an important role in the susceptibility to PEA-induced epatotoxicity that may depend on immune-regulation but not LRP receptor levels.

## 1. Introduction

*Pseudomonas aeruginosa* is an opportunistic, non-fermentative, Gram-negative rod bacterium. *P. aeruginosa* has emerged as a major infectious disease agent, particularly in patients with burn injuries or cystic fibrosis [1,2]. Several virulence factors, such as *P. aeruginosa* exotoxin A (PEA) and exoenzyme S, are involved in the disease caused by this bacterium [3]. Previous studies indicated that the low-density lipoprotein receptor-related protein (LRP) functions as the receptor that PEA utilizes to gain access to mammalian cells [4]. In addition, the decreased expression of LRP may enhance macrophage and hepatocyte cell-line resistance to PEA induced cytotoxicity [5,6]. Laithwaite and collaborators reported that increased PEA sensitivity in BNL 1ME A7R.1 transformed hepatocytes was associated with increased functional cell surface LRP expression [7].

To date, PEA has been used to establish an experimental animal model for immune-mediated liver injury [8]. In this model, PEA induces an excessive activation of host immune cells (especially Kupffer cells and T cells) that secrete pro-inflammatory cytokines, such as tumor necrosis factor α (TNF-α), interleukin (IL)-2 and IL-6, resulting in hepatocyte damage [8]. In our previous studies, we treated Wistar and Long-Evans (LE) rats with 20 μg/kg PEA and showed that Wistar rats were more resistant to PEA-induced liver injury than LE rats [8,9]. In other liver injury models, C57BL/6 mice have been reported to be resistant to Concanavalin A (ConA)-induced hepatitis, while BALB/c mice were susceptible. The difference in susceptibility between these mouse strains was associated with the development of a hepatitis that depends on IFN-γ production levels (high in C57BL/6 and low in BALB/c) [10]. In acetaminophen-induced hepatotoxicity models, significant hepatic hemorrhage was observed in C3He/FeJ and CD-1 mouse strains, but not in the C57BL/6 mouse strain [11]. These observations could be explained by differences in cytochrome P450 2E1 (CYP2E1) expression in sinusoidal endothelial cells, which has been shown to correlate with the susceptibility to vascular injury and hemorrhage [12]. On the other hand, C57BL/6 mice exhibited high deviations in the development of steatosis and inflammation in response to diet-induced features of non-alcoholic/alcoholic fatty liver disease [13]. Surprisingly, CD-1 mice did not show significant features of fatty liver disease in response to dietary regimens.

Previous studies have observed the development of severe, chronic asthma in a susceptible Brown Norway (BN) rat strain (BN/SsN), but not in a non-susceptible Fischer 344 (F344) strain [14,15]. Hines et al. indicated that pronounced macrophage and mast cell responses developed and persisted in BN/SsN, but not in F344 rats [15]. Although the genetic background is expected to affect the PEA-induced liver damage in different rat strains, this issue has not been sufficiently addressed in the literature. Thus, the current study was conducted to determine the differences in sensitivity to PEA-induced hepatotoxicity between four different inbred rat strains and two outbred stocks, namely F344, Wistar Kyoto (WKY), BN/SsN, Lewis (LEW), LE and Wistar by evaluating their clinical chemistry, liver histopathology and TUNEL staining. Moreover, in the liver LRP protein levels and quantification of pro- and anti-inflammatory cytokines in the serum were performed to determine if the levels of LRP receptors or inflammatory cytokines correlate with the differences in sensitivity to PEA-induced liver injury observed between rat strains.

## 2. Results

### 2.1. Hepatic Concentration of LRP in Different Rat Strains

**Figure 1:**
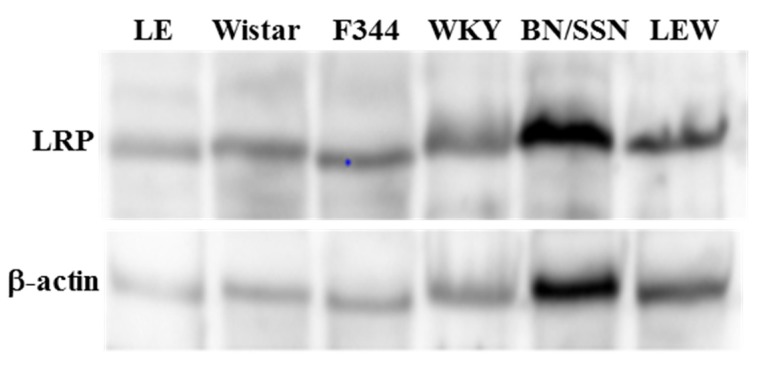
Protein levels of hepatic LRP in different rat strains. The membrane was stripped and immunoblotted with anti-β-actin antibody a protein loading control.

In order to elucidate the underlying mechanisms for PEA-hepatotoxicty in different rat strains, we reasoned that disease susceptibility may be related to differences in LRP expression or immune-regulation. We firstly evaluated the hepatic expression of LRP in different rat strains. However, no significant difference in LRP expression levels were observed in liver extract from each rat strain without PEA treatment (Figure 1).

### 2.2. Clinical Chemistry and Complete Blood Count (CBC) Analysis

**Figure 2:**
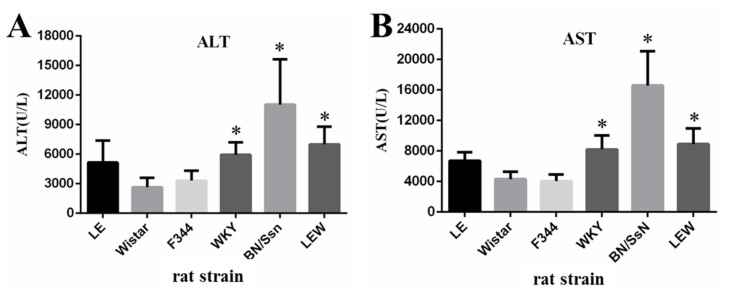
Serum levels of (**A**) ALT and (**B**) AST in the PEA-treated different rat strains. All rat strains including LE, Wistar, F344, WKY, BN/SsN and LEW were given PEA intravenously a dose of 20 μg/kg at 48h. Results are expressed as mean ± SD. * *p* < 0.05 compared with Wistar rats. Each group animal number = 8.

According to our previous report, the administration of 20 μg/kg PEA did not induce death of young adult Wistar and LE rats within the first 48 h post administration [8,9]. Thus, 48 h after PEA treatment, the serum ALT levels in LE, Wistar, F344, WKY, BN/SsN and LEW rats were analyzed and determined to be 5149 ± 2230.1 U/L, 2649 ± 937.2 U/L, 3312 ± 1022.6 U/L, 5124 ± 1255.1 U/L, 11032 ± 4587.2 U/L and 6992 ± 1788.4 U/L, respectively (Figure 2). Moreover, the fold changes of ALT compared with basal levels of each rat strains were 88 ± 34.1, 49.2 ± 16.1, 50.7 ± 14.3, 87.7 ± 16.6, 180 ± 68.3 and 127.4 ± 29.1, respectively (Figure S1).

A similar trend, as well as fold changes in the serum levels for AST, were also observed between the different rat strains. Notably, ALT and AST serum concentrations were significantly greater in BN/SsN rats than in other species, while the lowest serum levels were observed in Wistar and F344 rat strains. 

**Figure 3:**
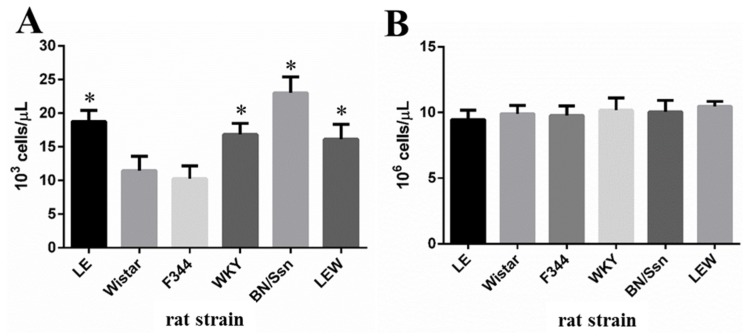
Effect of 20 μg/kg PEA on hematological characteristics of different rat strains (**A**) WBC; (**B**) RBC. Results are expressed as mean ± SD. * *p* < 0.05 compared with Wistar rats.

As shown in Figure 3, the total white blood cell (WBC) count was markedly increased in the BN/SsN group compared to that in other groups. Meanwhile, total WBC counts were lower in the LE, WKY, and LEW rats compared to those in BN/SsN rats, but still higher than those for Wistar and F344 rats. In comparison, no significant differences between groups were observed for the total red blood cell (RBC) counts. The treatment of all rat strains with Dulbecco’s phosphate-buffered saline (DPBS) did not cause ALT and AST elevations, and showed no significant changes in CBC (Figure S1, Table S1).

### 2.3. Histopathology and TUNEL Staining

**Figure 4:**
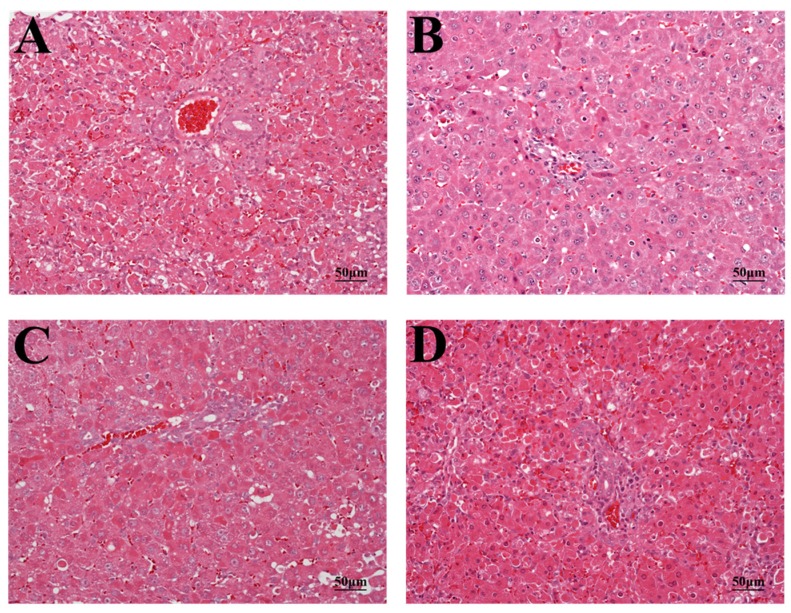

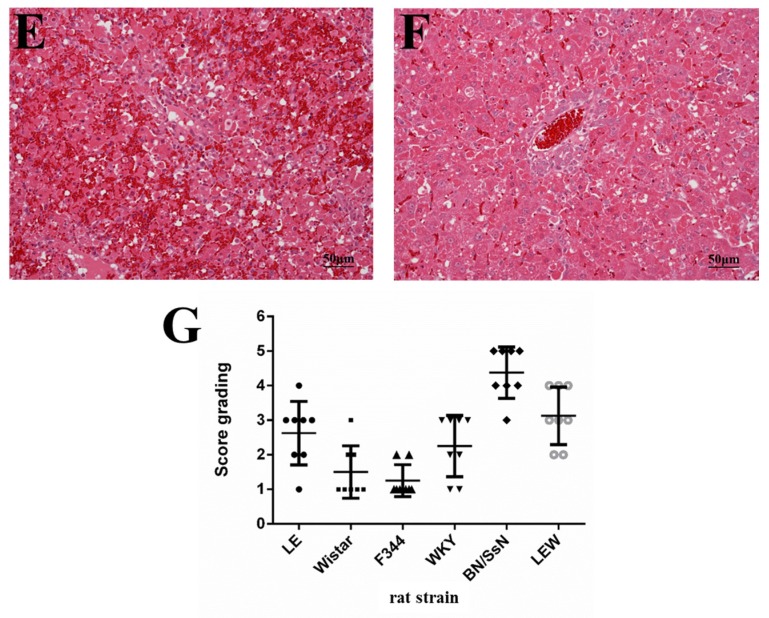
The photomicrographs of liver tissues from rats treated with 20 μg/kg PEA at 48 h. (**A**) LE; (**B**) Wistar; (**C**) F344; (**D**) WKY; (**E**) BN/SSN; (**F**) LEW; (**G**) Histologic score was combined hepatic lesions and hemorrhage grading (mean ± SD). H&E stain, magnification ×200.

Analysis of histologic liver sections from PEA-treated BN/SsN rats showed diffuse, massive necrosis, hepatocyte nuclear condensation, hemorrhage, and dissociation of hepatic cords. In sections of LE, WKY and LEW rats, numerous apoptotic bodies and moderate hepatocyte necrosis were observed. Interestingly, only a small number of apoptotic bodies and mild hepatocyte necrosis were found in histological sections of Wistar and F344 rats (Figure 4). The mean liver injury scores (on a scale from 0 to 5) were calculated for the different rat strains and determined to be for LE: 2.63 ± 0.92, Wistar: 1.51 ± 0.76, F344: 1.25 ± 0.46, WKY: 2.25 ± 0.88, BN/SsN: 4.37 ± 0.74 and LEW: 3.12 ± 0.83.

**Figure 5:**
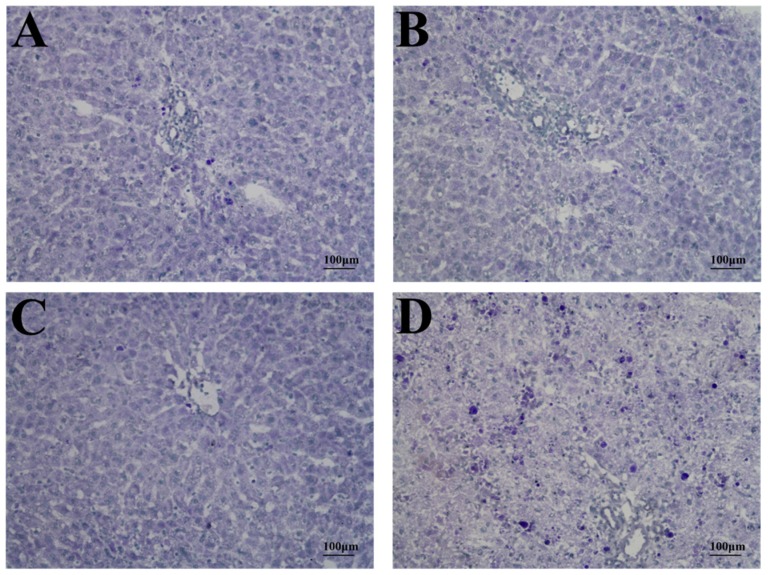

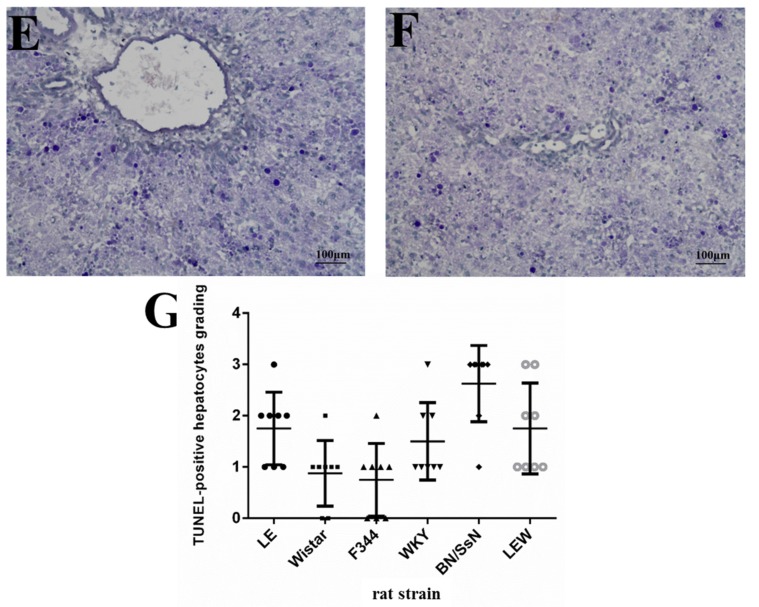
Evaluation of apoptotic hepatocytes by TUNEL assay in the PEA-treated different strain rats at 48 h. (**A**) LE; (**B**) Wistar; (**C**) F344; (**D**) WKY; (**E**) BN/SSN; (**F**) LEW; (**G**) TUNEL score, which was dependent on the number of TUNEL-positive cells counted *per* 100× field (mean ± SD). TUNEL stain, magnification ×200.

Treatment with 20 μg/kg PEA caused the presence of a significant number of apoptotic nuclei in the livers of BN/SsN rats. In contrast to BN/SsN rats, the numbers of TUNEL-positive cells were markedly lower in liver sections of LE, WKY, and LEW rats. Only a few TUNEL-positive cells were observed in liver sections of Wistar and F344 rats (Figure 5). The average number of TUNEL-positive hepatocytes (per 100× field) present were calculated to be for LE: 1.62 ± 0.71, Wistar: 0.87 ± 0.64, F344: 0.74 ± 0.69, WKY: 1.53 ± 0.75, BN/SsN: 2.67 ± 0.74 and LEW: 1.75 ± 0.88. No histologic changes or TUNEL-positive cells were observed in the liver samples from DPBS-treated different rat strains (Table S1).

### 2.4. Serum Cytokine Levels

**Figure 6:**
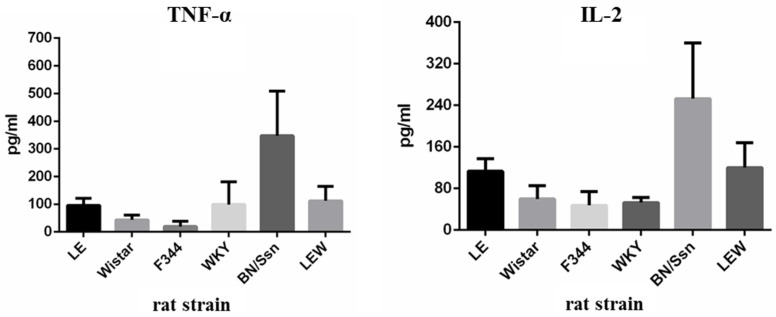

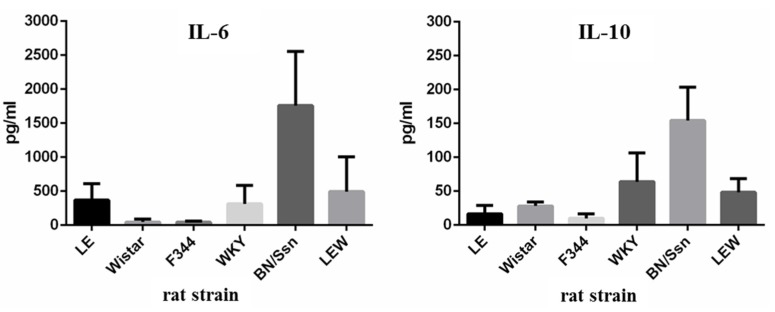
Serum cytokine profiles, including TNF-α, IL-2, IL-6 and IL-10 levels from rat strains challenged with 20 μg/kg PEA for 48 h**.** Results are expressed as mean ± SD.

Serum cytokine levels are summarized in Figure 6. BN/SsN rats had significantly higher TNF-α serum levels compared to those in other rat strains. Moderate TNF-α serum levels were observed in LE, WKY and LEW rats. Only trace amounts of TNF-α were detected in F344 rats. The highest levels of serum IL-2 were found in BN/SsN rats. Serum IL-2 concentrations in Wistar, F344, and WKY rats were significantly lower than those in BN/SsN rats. In LE and LEW rats, the levels of serum IL-2 were moderate. Levels IL-6 in serum were significantly higher in BN/SsN rats compared to those in the LE, WKY and LEW rats. Interestingly, only low levels of IL-6 were detected in Wistar and F344 rats. BN/SsN rats exhibited significantly higher IL-10 levels in serum when compared to other groups. Meanwhile, the lower levels of IL-10 were noted in F344 rats. Serum TNF-α, IL-2, IL-6, and IL-10 were not detected in any of the DPBS-treated rat strains (Table S1).

## 3. Discussion

The objective of this investigation was to better understand different stocks (LE and Wistar) and strains (F344, WKY, BN/SsN and LEW) of rats in their susceptibility or resistance to a sub-lethal dose of PEA-induced liver injury. The present study showed that BN/SsN rats had severe liver injury in response to treatment with a sub-lethal dose of PEA, while moderate liver damage developed in LE, WKY, and LEW rats. In contrast, PEA treatment induced only low numbers of apoptotic and necrotic hepatocytes in both Wistar and F344 rats. These differences in hepatotoxicity are observed not only with a sub-lethal dose of PEA as shown herein, but also with other mechanistically different models of T-cell-dependent liver injury after treatment with Concanavalin A, acetaminophen or lipopolysaccharide (LPS) treatment [10,11,16,17]. Importantly, these results can serve as reference data relating to PEA treatment in different rat genetic backgrounds, which may prove useful for PEA-based anti-tumor drug development.

The present results revealed significant differences in the response of several rat strains to PEA administration. Clinical chemistry values in the blood for ALT and AST were significantly higher in BN/SsN rats compared with other strains. The lowest levels of ALT and AST were noted in Wistar and F344 rats. On the other hand, morphological features in of liver damage that included hepatic cord dissociation, liver hemorrhage, and massive hepatocytes necrosis were apparent in BN/SsN rats. A moderate level of hepatocyte necrosis and apoptosis were observed in LE, WKY and LEW rats, whereas only a small number of apoptotic bodies and single cell necrosis were observed in Wistar and F344 rats. These findings are similar to those of previous reports, which show differences in PEA-induced hepatotoxicity in different mouse strains [18,19,20]. Our data suggests that BN/SsN rats were more sensitive to PEA treatment than Wistar and F344 strains. Although the only one dose (20 μg/kg) and time point (48 h) was performed in these different rat strains. However, our previous results found that the severity of PEA-induced liver injury was dose and time dependent in Wistar and LE rats [8,9].

We have previously demonstrated that Kupffer cells are capable of producing large amounts of TNF-α, and thus can cause severe liver injury, suggesting an important role for hepatic Kupffer cells in PEA-induced hepatotoxicity [8]. It has been shown that gadolinium chloride (GdCl3) can ameliorate pro-inflammatory cytokine production, as demonstrated in our previous study [21]. In this study, among all the rat strains tested, the highest levels of TNF-α were detected in BN/SsN rats. This finding is consistent with that of our previous studies, which indicated that the severity of PEA-induced hepatotoxicity was closely correlated with pro-inflammatory cytokines such as TNF-α [8,9]. 

A previous study has also shown that IL-2 stimulated the expression of pro-inflammatory cytokines/chemokines from liver nonparenchymal cells, such as T cells, Kupffer cells, and stellate cells, which contributes to liver inflammation [19]. The role of IL-2 was believed to be a pivotal mediator of liver injury in PEA-treated rats [8,18,19]. The present study showed that the PEA-induced serum levels of IL-2 directly correlated with the severity of hepatotoxicity, i.e., IL-2 values were the highest in BN/SsN, intermediate and variable in LE, WKY and LEW, and lowest in Wistar and F344 rats. This phenomenon showed that PEA treatment caused different levels of hepatotoxicity in different rat strains. In Con-A induced liver injury models, increased plasma IL-2 levels and T cell infiltration were observed [10,22]. Therefore, inhibition of the IL-2 signaling pathway might offer protection from this hepatotoxic effect [23]. A recent study has shown that inhibition of T cells producing IL-2 may prevent PEA-induced hepatotoxicity [19]. Therefore, we suggest that PEA-induced hepatotoxicity in different rat strains directly correlates with the capacity of IL-2 induction.

IL-6 is a cytokine involved not only in inflammation and infection responses, but also in the regulation of metabolic, regenerative, and neural processes [24]. In murine sepsis models, blockade of IL-6 trans-signaling was sufficient to rescue mice from death [25]. Blockade of IL-6 signaling aggravates liver injury and lethality in a D-galactosamine N (GalN)/LPS hepatitis model [26]. In our study, serum IL-6 levels were significantly higher in the BN/SsN rats, but it was rarely detected, or detected at very low concentrations in Wistar and F344 strain rats. This phenomenon was consistent with our previous studies, indicating that the IL-6 dependent signal pathway may be involved in immune-mediated, PEA-induced hepatotoxicity.

IL-10 is a potent anti-inflammatory cytokine that inhibits the inflammatory reaction, initiation, and progress [27]. In this work, the serum levels of IL-10 increased significantly in the BN/SsN rats compared with other rat strains. This is presumably a consequence of the severe liver injury induced by PEA in BN/SsN rats. In contrast, the serum levels for IL-10 were low to moderate in other rat strains. Therefore, these results directly correlate with the degree of liver tissue injuries observed between the different rat strains.

Previous studies indicated the absence or reduce of LRP in different cell line, including LRP^-/-^ embryonic fibroblasts, hepatoma and macrophage, were sufficient to protect cells from PEA intoxication [4,5,6,7]. These findings suggested that PEA might use LRP to gain entry into toxin-sensitive cells. In the present study, we compared the LRP expression in liver tissue from different rat strains. However, the results showed no significantly difference in LRP expression levels in each group. Based on these results, we suspect that the PEA-mediated differences in severity of liver injury we detected in different rat strains may not correlated with LRP expression.

In conclusion, we have demonstrated the differences in the genetic susceptibility to PEA-induced liver injury among four inbred strains (F344, WKY, BN/SsN and LEW) and two stocks (LE and Wistar) of rats, and found BN/SsN rats to be the most sensitive. Our findings suggest that the genetic background plays an important role in the risk for hepatotoxicity in immune-mediated liver injury. In addition, strain differences in the susceptibility of PEA-induced liver injury may be influenced by the response to pro-inflammatory cytokines (TNF-α, IL-2 and IL-6) that are induced as part of the inflammation process, which is in turn determined by the genetic background. Due to this, PEA-based immunotoxins for clinical use are being developed by many research teams [28,29]. Our data support the notion that variations in the immune response may be the most critical factor in PEA-induced hepatotoxicity. Hence, these results support the concept that variations in the genetic background of the host will manifest as different responses to diverse xenobiotic or biohazard intoxication.

## 4. Materials and Methods 

Chemical and Animals. PEA was purchased from Calbiochem Chemical Company (La Jolla, CA, USA). A total of 72 specific-pathogen-free male LE, Wistar, F344, WKY, BN/SsN and LEW rats were purchased from BioLASCO Technology (Taipei, Taiwan) and National Laboratory Animal Center (Taipei, Taiwan), and used at 8–9 weeks of age. Animals were maintained at room temperature (21 ± 2 °C), with 55–65% relative humidity, and a 12 h light-dark cycle. Rats were fed a standard laboratory rat diet and provided with water *ad libitum*. All procedures were performed in an animal facility accredited by the Association for Assessment and Accreditation of Laboratory Animal Care (AAALAC) International, with the approval of the Institutional Animal Care and Use Committee (IACUC)(Ethical approval code: NLAC-94-R-001, Date of approval: 28 December 2005).

Experimental procedures. Different rat strains (eight per strain) were assigned to six groups. Rats were injected intravenously via the tail vein with 20 μg/kg PEA diluted in Dulbecco’s phosphate-buffered saline (DPBS) (Gibco, Grand Island, NY, USA). For the purpose of minimizing animal use, the DPBS control groups contained four rats *per* strain for each rat strain. After 48 h, the animals were euthanized by CO2 asphyxiation followed by exsanguination. Blood was withdrawn by cardiac puncture for analysis of clinical chemistry, complete blood count (CBC), and quantification of cytokines. Livers were excised and divided into several parts for various assays.

Clinical chemistry in blood and CBC. Serum samples were centrifuged at 2700 g for 10 min. Liver function tests, including serum aspartate aminotransferase (AST), alanine aminotransferase (ALT), and icterus index were done using an automatic analyzer (HITACHI 717, Hitachi, Tokyo, Japan). Blood used for CBC was treated with EDTA. Total blood cells, differential leukocytes, and erythrocytes were measured using the Bayer Hematology System (ADVIA 2010, Bayer, NY, USA).

Histological examination and TUNEL staining. Liver fixation, processing, and embedding were performed as previously described [9]. Sagittal sections of the left lobe were obtained and stained with hematoxylin and eosin (H&E) for histological examination. The scores, grading, and definition of hepatic lesions were: 0 = no lesions, no necrosis; 1 = mild, single-cell necrosis; 2 = moderate, hepatocyte necrosis mostly around periportal areas; and 3 = severe, extensive to massive necrosis [21]. The scoring and grading for hemorrhage was: 0 = no hemorrhage; 1 = mild hemorrhage; and 2 = severe hemorrhage. The results of score was combined hepatic lesions and hemorrhage grading.

For the detection of hepatocyte apoptosis, 4 mm paraffin-embedded sections were deparaffinized, rehydrated, and stained with an apoptosis-specific staining kit (In Situ Cell Death/AP Detection Kit; Roche Diagnostics, Mannheim, Germany) according to the manufacturer’s instructions. Slides were randomized, coded, and evaluated by a veterinary pathologist using light microscopy. The scores for the level of apoptosis were dependent on the number of TUNEL-positive cells *per* 100× magnification field: 0 = no TUNEL-positive cells; 1 = 2–5 TUNEL-positive cells; 2 = 6–10 TUNEL-positive cells; and 3 = more than 11 TUNEL-positive cells. This calculation method has been described previously.

Serum cytokine levels assay. Quantitative determinations of serum levels for TNF-α, IL-2, IL-6 and IL-10 were performed using a specific ELISA kits (Invitrogen, Carlsbad, CA, USA). The ELISA assays were performed as detailed by the manufacturer.

Measurement of hepatic LRP expression levels. The liver tissue was homogenized immediately in extraction buffer using a handheld homogenizer (T 10 basic, IKA, Germany). The extracts were centrifuged at 11,000 rpm and 4 C in a Sigma Centrifuge 4k15 (Ostrode am Harz, Germany) for 30 min to remove insoluble material, and the supernatants of these tissues were used for protein quantification, using the Bradford method. Aliquots of 0.2 mg of protein extracts, obtained from each tissue, were separated by SDS-PAGE, transferred to nitrocellulose membranes and blotted with anti-LRP and anti-β-actin antibodies, from Santa Cruz Biotechnology (Santa Cruz, CA, USA). The original membrane was stripped and reblotted with β-actin loading protein. Chemiluminescent detection was performed with horseradish peroxidase-conjugate secondary antibodies. Visualization of protein bands was performed by exposure of membranes to RX films. Band intensities were quantified by optical densitometry (Scion Image software, version 4.0.3, ScionCorp, Frederick, MD, USA) of the developed autoradiographs.

Statistical analysis. Data including clinical chemistry and serum cytokines were presented as mean ± standard deviation (mean ± SD). Significant differences between each treated group were determined by one-way analysis of variance (ANOVA) and post hoc Fisher’s least significant differences (LSD) test by SPSS 18.0 software. Differences between groups were considered to be statistically significant (*) when *p* < 0.05. 

## Supplementary Materials

The following are available online at www.mdpi.com/2072-6651/9/7/224/s1, Figure S1. The expression fold change of the (A) ALT and (B) AST in the PEA-treated rat compared to DPBS-treated rat, Table S1. Clinical chemistry, complete blood count, cytokines of serum and score grading in the DPBS-treated rats.
