# Supplementary material for: Differences in Genetic Background Contribute to Pseudomonas Exotoxin A-Induced Hepatotoxicity in Rats
Source: Toxins (Basel). 2017 Jul 15;9(7):224. doi: 10.3390/toxins9070224 (PMC5535171; doi:10.3390/toxins9070224)
Supplement: Supplementary file 1 [file toxins-09-00224-s001.pdf]

Supplementary Materials: Differences in Genetic Background Contribute to *Pseudomonas* Exotoxin A-Induced Hepatotoxicity in Rats

Chien-Chao Chiu, Yu-Chih Wang, Wen-Ching Huang, Yi-Hsun Chen, Shao-Wen Hung, Yen-Te Huang, Hsiao-Li Chuang and Yi-Chih Chang

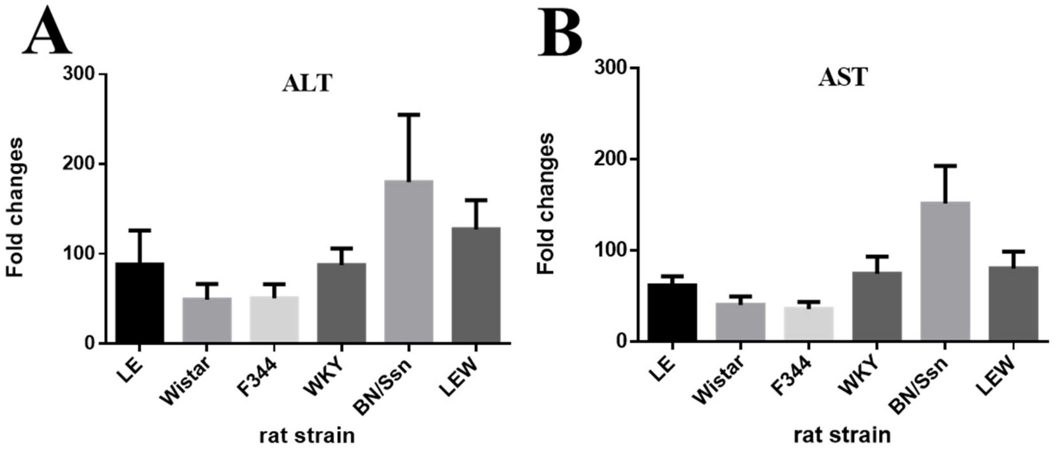

Figure S1. The expression fold change of the (**A**) ALT and (**B**) AST in the PEA-treated rat compared to DPBS-treated rat. Results are expressed as mean ± SD. Each group animal number=8.

Table S1. Clinical chemistry, complete blood count, cytokines of serum and score grading in the DPBS-treated rats.

Parameters/Rat strain	LE	Wistar	F344	WKY	BN/Ssn	LEW
Clinical chemistry						
ALT (U/L)	58.5 ± 7.8	53.8 ± 9.3	65.3 ± 13.2	67.7 ± 9.9	61.3 ± 7.9	54.9 ± 11.3
AST (U/L)	108.5 ± 15.1	106.3 ± 21.2	112.0 ± 19.3	108.3 ± 11.1	109.2 ± 15.1	110.5 ± 14.9
CBC*						
WBC (10^3 cell/μl)	8.9 ± 1.2	9.0 ± 2.2	8.5 ± 1.3	8.7 ± 1.5	9.1 ± 2.6	8.5 ± 1.5
RBC (10^6 cell/μl)	7.6 ± 0.8	8.5 ± 0.7	7.5 ± 1.3	7.9 ± 1.0	8.9 ± 1.5	8.5 ± 1.1
Cytokines						
TNF-α (pg/ml)	ND[#]	ND	ND	ND	ND	ND
IL-2 (pg/ml)	ND	ND	ND	ND	ND	ND
IL-6 (pg/ml)	ND	ND	ND	ND	ND	ND
IL-10 (pg/ml)	ND	ND	ND	ND	ND	ND
H&E staining						
Score grading[$]	0 ± 0	0 ± 0	0 ± 0	0 ± 0	0 ± 0	0 ± 0
TUNEL staining						
TUNEL-positive hepatocytes grading[@]	0 ± 0	0 ± 0	0 ± 0	0 ± 0	0 ± 0	0 ± 0

*: CBC, complete blood count; [#]: ND, Not detected; [$]: Score grading; 0 = no lesions, no necrosis; 1 = mild, single-cell necrosis; 2 = moderate, hepatocyte necrosis mostly around periportal areas; and 3 = severe, extensive to massive necrosis. [@]: TUNEL-positive hepatocytes grading, 0 = no TUNEL-positive cells; 1 = 2–5 TUNEL-positive cells; 2 = 6–10 TUNEL-positive cells; and 3 = more than 11 TUNEL-positive cells.
